# Synergistic approach to patient dialysate

**Published:** 2015

**Authors:** A Dragotoiu, AI Checheriţă, A Ciocâlteu, S Rizeanu

**Affiliations:** * Center for Dialysis, Slatina, Olt, Romania; ** “Sfantul Ioan” Nephrology Clinic, Bucharest, Romania; “Carol Davila” University of Medicine and Pharmacy, Bucharest, Romania; *** Aquamarin Clinic, Bucharest, Romania

**Keywords:** synergetic, stress, resilience, dialysis, psychic

## Abstract

The stress a patient is subjected to during dialysis treatment can be reduced by using a synergetic approach by the medical team. The integration into therapy of the positive psychical resources such as: active positive coping mechanisms, individual or family mental resilience, improvement of the image and self-esteem, better tolerance to frustration can represent an important part in the improvement of the patient’s quality of life, determination of a positive approach of the situations both for him and close friends and relatives.

The long-term positive expectancies of the patient dialysate, the more complex integration in his social environment and a better condition, represent some objectives which any medical team which treats dialyzed patients establishes. 

The synergistic approach of the patient dialysate represents a method which allows the integration of psychology elements that help in a faster accomplishing of the proposed objectives, in the specialty treatment. 

Synergetics refers to the simultaneous action of more agents, even of different kinds, in order to perform the same function. It concentrates on studying, auto organizing or auto structuring the systems, no matter their nature (chemical, physical, biological or social), starting from the total cooperation of all the components and constitutive systems and having as a final purpose the performance of the same function. The cooperation of these elements finally leads to the obtaining of some remarkable effects both at the macro social and at the micro social level [**1**,**2**,**9**]. 

Objectively, synergetics approaches the following types of issues: 

-auto organizing which is done by a synchronous cooperation of all the constituents 

-the appearance of order out of chaos 

-the phase transitions of the system by overcoming a critical threshold

-the problem of chaos which finally generates order.

All these elements lead to the conclusion that a synergetic system, among the characteristics and properties which all the systems generally have (such as dynamics, organization, interaction, finality), also has an extra characteristic: synergy, meaning the global effect of cooperation of the sides (subsystems, components) and their use for the whole. 

In this case we are talking about the so-called efficient synergy which does not imply only a quantitative growth of the components but also a qualitative growth resulted from the fine-tuning elements of the system. 

Paradoxically, disorder is not only destructive but also has the ability of resetting the whole, often being able to create a new order. Often, disorder appears as an absolutely necessary moment in an upward development process. Order and disorder cannot be regarded as being opposed to one another but rather complementary. That is why both dysfunctional order and functional order exist and act permanently. 

In the synergetic systems, order generates disorder and vice-versa, the organization is responsible for balance, regulation and autoregulation, and, the auto organization assures the transition from balance to controlled unbalance, thus avoiding the degeneration of the system. 

The effects of auto organization specific to living systems, which have the capacity of learning and also have a conscience, allow the growth of the degree of initiative, autonomy , freedom and creation of a person. Synergetics represents the most advanced theory of auto organization, this being specific to living structures and absent in artificial structures, trying to highlight the growth of the efficiency of the system by optimizing the whole with the help of the internal cooperation mechanism of the components [**8**-**10**]. 

The interest for an optimum auto organization which can be calculated and controlled, corroborated with the capacity of the element which works for the whole, shows that synergetics overcomes the general theory of the systems, this paradigm being able to underlie the new research or practical approach methods of any type of problem. 

What should be mentioned is that in the functioning of the synergetic system, the combination, cooperation and counteracting of the elements is very important, underlining the fact that without this collaboration, such a system could lose its dynamism and complexity which characterizes it, and finally it could also lose its role as a system.

With regard to this it should be mentioned that synergetics opens new horizons to psychological research, not only in the direction of opening and explaining but also in the direction of human construction, allowing an easier cooperation between the theoretical and the experimental approach [**1**-**3**]. 

## The psychic problem in the patient dialysate 

Dealing with hard situations and the need to solve them permanently appeals to the area of human behavior, the main goal of the human cognitive system being the one of solving problems.

Usually, the chronic patient and especially the patient dialysed has some needs regarding the way he understands the pathology he suffers from and the way he adapts to the treatment specific for his disease [**4**-**7**]. 

In order to find an efficient resolution strategy, the patient should find as many ways of solving the problem, thus being able to simultaneously deal with both the advantages and especially the limits of each therapeutic method. This way he will know that there are many alternatives of solving his problem, by accepting the positive sides and the negative sides also of the proposed solutions. 

The life of the patient dialysed presents many constraints which should be excluded by offering some models or methods of approaching this problem, trying to improve the quality of life and also the growth of compliance to the treatment specific to the disease. 

The synergetic approach could help the patient solve a wide range of tasks, allowing him to overcome the feeling of social usefulness or of permanent burden for everybody around by improving two of the most important components of his personality: self-esteem and self-image (the way he sees himself). The trust in his own physical and psychic resources allows a more adequate reporting to the task, this way having the chance that through this type of approach, we could obtain a wellbeing extended on a larger period of time in the life of the patient. 

A problem becomes acute or obvious when a person proposes to have an aim or faces the situation of reacting to a stimulus, for which he does not have an adequate answer stored in his memory. 

Certainly, we say we are dealing with a problem when a desirable situation differs from the initial one, which the person is motivated to solve, combines with lots of actions or operations which finally lead to the achievement of the goal. 

In the case of the patient dialysate, the psychic problem is reduced to the improvement of the attitude towards the treatment specific to dialysis, as far as the medical team and the support group are concerned, by optimizing not only the psychic resilience but also the coping mechanisms through a synergic approach of these components. 

Patients who manage to change their negative thinking will have a better self-image and self-esteem, will go through less anxiety states, depression or frustrations and will easily engage in social relationships specific to their interests, generating a better performance in their daily activities.

Objectively, they will have to learn that despite their beliefs regarding the validity of the negative thoughts, most of them are distorted and very unrealistic. Obviously, there are situations in which negative thoughts can be real, but in this case they will have to adapt and accept these states of discomfort and in the same time learn to cope with them. 

There are lots of types of distortions in case of a patient dialysate:

-Overgeneralization – this type of distortion appears when a negative event which happens to some dialysis patients whom he knows is considered by the patient that will certainly happen to him. 

-False predictions regarding the future – this kind of distortion is associated by the patients with the induction of a negative state starting from unrealistic presuppositions. 

-Amplification (of certain states) – is produced when the patients exaggerate regarding the importance and intensity of pains in certain cases, the result being unjustified modifications of the treatment. 

-Negative mental filtering – appears when the patients choose a negative event and they only concentrate on it, in such a way that the whole reality becomes distorted. 

-Positive neglect – this kind of distortion is produced when the patient refuses to accept any positive element in his life, excluding any state of wellbeing and focusing exclusively on his status of patient dialysate. 

-Drawing some wrong conclusions – represents the tendency of reaching a conclusion without having enough elements of analysis on some events which have a negative tendency during their evolution. 

-Labeling – represents that way of thinking in which the patient labels himself but also the persons around him according to his affective state without appealing to rational thinking elements, inducing a distorted behavior as far as he or the others are concerned [**5**,**6**,**8**].

All these thoughts and irrational beliefs can be corrected by identifying the distortions, being aware of their existence, accepting the shades and alternatives and then, by reevaluating the thoughts and feelings which the patient deals with in that context regarding that certain person. 

Individual neurosis can easily turn into psychosis and the fixation which leads to this state can rapidly be transmitted to the whole group of patients dialysate, thus altering the process of treatment

Going through the physical state, the patient dialysate must handle an absolute dependency psychic state, which makes his access to his basic resources harder, the dependency being already a part of the disease.

The synergistic approach represents a faster way of access to the resources of the patient (coping mechanisms, psychic resilience, tolerance to frustration, etc.), but also his better integration in his social environment [**4**,**7**,**9**,**10**].
